# Emotional Attitudes of Chinese Citizens on Social Distancing During the COVID-19 Outbreak: Analysis of Social Media Data

**DOI:** 10.2196/27079

**Published:** 2021-03-16

**Authors:** Lining Shen, Rui Yao, Wenli Zhang, Richard Evans, Guang Cao, Zhiguo Zhang

**Affiliations:** 1 School of Medicine and Health Management Tongji Medical College Huazhong University of Science & Technology Wuhan China; 2 Hubei Provincial Research Center for Health Technology Assessment Wuhan China; 3 Institute of Smart Health Huazhong University of Science & Technology Wuhan China; 4 College of Engineering Design and Physical Sciences Brunel University London London United Kingdom

**Keywords:** COVID-19, Sina Weibo, social distancing measures, emotional analysis, machine learning, moderating effects, deep learning, social media, emotion, attitude, infodemiology, infoveillance

## Abstract

**Background:**

Wuhan, China, the epicenter of the COVID-19 pandemic, imposed citywide lockdown measures on January 23, 2020. Neighboring cities in Hubei Province followed suit with the government enforcing social distancing measures to restrict the spread of the disease throughout the province. Few studies have examined the emotional attitudes of citizens as expressed on social media toward the imposed social distancing measures and the factors that affected their emotions.

**Objective:**

The aim of this study was twofold. First, we aimed to detect the emotional attitudes of different groups of users on Sina Weibo toward the social distancing measures imposed by the People’s Government of Hubei Province. Second, the influencing factors of their emotions, as well as the impact of the imposed measures on users’ emotions, was studied.

**Methods:**

Sina Weibo, one of China’s largest social media platforms, was chosen as the primary data source. The time span of selected data was from January 21, 2020, to March 24, 2020, while analysis was completed in late June 2020. Bi-directional long short-term memory (Bi-LSTM) was used to analyze users’ emotions, while logistic regression analysis was employed to explore the influence of explanatory variables on users’ emotions, such as age and spatial location. Further, the moderating effects of social distancing measures on the relationship between user characteristics and users’ emotions were assessed by observing the interaction effects between the measures and explanatory variables.

**Results:**

Based on the 63,169 comments obtained, we identified six topics of discussion—(1) delaying the resumption of work and school, (2) travel restrictions, (3) traffic restrictions, (4) extending the Lunar New Year holiday, (5) closing public spaces, and (6) community containment. There was no multicollinearity in the data during statistical analysis; the Hosmer-Lemeshow goodness-of-fit was 0.24 (*χ*^2^_8_=10.34, *P*>.24). The main emotions shown by citizens were negative, including anger and fear. Users located in Hubei Province showed the highest amount of negative emotions in Mainland China. There are statistically significant differences in the distribution of emotional polarity between social distancing measures (*χ*^2^_20_=19,084.73, *P*<.001), as well as emotional polarity between genders (*χ*^2^_4_=1784.59, *P*<.001) and emotional polarity between spatial locations (*χ*^2^_4_=1659.67, *P*<.001). Compared with other types of social distancing measures, the measures of *delaying the resumption of work and school* or *travel restrictions* mainly had a positive moderating effect on public emotion, while *traffic restrictions* or *community containment* had a negative moderating effect on public emotion.

**Conclusions:**

Findings provide a reference point for the adoption of epidemic prevention and control measures, and are considered helpful for government agencies to take timely actions to alleviate negative emotions during public health emergencies.

## Introduction

### Background

In late 2019, COVID-19 began to spread rapidly throughout Hubei Province, China, creating devastating consequences for citizens and organizations and heavily burdening the provision of public health care in the province. The unknown pneumonia strain led to a major public health emergency; it has been deemed to be the disease with the fastest transmission rate, the greatest infection rate, and the most difficult to prevent since the establishment of New China [1]. The World Health Organization declared COVID-19 a public health emergency of international concern on January 31, 2020, and a pandemic on March 11, 2020 [2]. To prevent further outbreaks and disease transmission, the Chinese government adopted a series of measures to restrict the transmission and infection of the disease during the Lunar New Year holiday [3]. Although the 2020 Chinese Lunar New Year holiday is from January 24–31, 2020, to prevent and control the epidemic, the General Office of the State Council extended the holiday to February 2, 2020. At the epicenter of the epidemic, the People’s Government of Hubei Province extended the holiday to February 13, 2020. The most widely imposed measure was to increase physical distancing between people [4], including traffic restrictions [5], delaying the resumption of work and school [6], extending the Lunar New Year holiday [7], travel restrictions [8], and community containment and closing of public spaces [9]. Such measures that aim to reduce exposure to the disease, by reducing contact between people, is known as “social distancing” [10]. The United States Centers for Disease Control and Prevention defined social distancing as the restriction of close face-to-face contact with others and considers it the best method for reducing the spread of COVID-19 [11].

Since the advent of the internet, social media has become an indispensable part of citizens’ lives, greatly enriching the way people share feelings and exchange opinions. As of October 2020, there were approximately 4.66 billion active internet users worldwide, including 4.14 billion active social media users, accounting for 59% and 51% of the global population, respectively [12]. According to the 46th China Statistical Report on Internet Development, revised in June 2020, the number of Chinese internet users has reached 940 million [13]. International social media users tend to express their opinions on Twitter, due to its quick release and acceptance of information [14]. As an alternative to Twitter in Mainland China, Sina Weibo [15] has played an important role during many public health emergencies in recent years [16]. Studies have shown that through the analysis of content published on social media platforms, citizens’ views and attitudes toward an event can be tracked and discovered [17,18]. During the COVID-19 pandemic, netizens expressed significant views on the imposed social distancing measures for disease prevention and control in Hubei Province. Through the collection of text related to social distancing on the internet, we can gain a better understanding of Sina Weibo users’ emotional attitudes toward the imposed measures, so as to provide data for government agencies to implement measures in a timely fashion.

### Related Work

#### Social Distancing During Public Health Emergencies

In public health emergencies, social distancing has become an important course of action to prevent the spread of diseases. For example, social distancing measures imposed during the influenza pandemic drastically reduced the infection rate [19,20]. One study into the severe acute respiratory syndrome (SARS) epidemic found that the prevention and control of the epidemic, through the implementation of social distancing measures, had a certain effect in Canada [21]. During the COVID-19 pandemic, government agencies have also encouraged the adoption of social distancing measures. Zhang et al [22] studied the impact of social distancing measures on the spread of COVID-19 by establishing a disease transmission model. Other studies have confirmed that by closing schools and universities, there has been a significant reduction in the spread of the disease [23-25]. Self-quarantine measures also helped reduce the transmission rates of influenza in Texas in 2009, as well as for SARS in Singapore in 2003 [26,27]. The sanitary cordon helped set back an epidemic of Ebola in 1995 in the city of Kikwit, Zaire [28]. Workplace social distancing guidelines delayed and reduced the peak influenza attack rate [29]. In addition, travel restrictions and the canceling of mass gatherings have also been effective strategies for reducing the burden of COVID-19 on health care providers [30,31].

However, the use of social distancing measures by local governments during the COVID-19 pandemic may have a negative impact on citizens’ mental health and well-being [32,33]. Therefore, it is important to study the psychology of citizens and their behaviors in the context of social distancing measures.

#### Emotional Expression During Public Health Emergencies

Emotional polarity analysis is often used to examine the emotional tendencies expressed in text and to discover the emotional attitudes of users. Some studies have found that the pandemic has led to negative emotions being expressed by internet users [34,35]. For example, Ogoina et al [36] found that health care workers demonstrated varying degrees of fear related to the Ebola epidemic in Nigeria in 2014, through self-administered questionnaires and documented observations. Other scholars have explored the emotional attitudes of citizens toward epidemic diseases through questionnaires [37,38]. However, all studies have only explored the change in citizens’ emotions through cross-sectional data, and the samples examined were not large.

As an important medium for public communication, social media provides a large-scale corpus for emotional analysis. Emotion lexicons and machine learning are two methods commonly used for analytical analysis. In terms of emotion lexicons, there are relatively mature lexical resources available, such as SentiWordNet and the National Research Council of Canada (NRC) word-emotion lexicon [39,40]. Das et al [41] used the NRC word-emotion lexicon to analyze the emotions of Twitter users about COVID-19 in India. In the field of machine learning, Du et al [42] used a convolutional neural network (CNN) classifier to conduct an emotional analysis on Twitter data during the measles outbreak. Ji et al [43] also found that multinomial naive Bayes (NB) was effective in analyzing the emotion of Twitter users facing epidemics. Although a classification method based on emotion lexicons is effective, it relies heavily on the scale and frequency of updating the lexicon, while a classification method based on machine learning avoids this limitation and is widely used today. For example, Behera et al [44] studied the emotional classification of tweets related to three diseases—malaria, swine flu, and cancer—through emotion lexicon and NB methods. Their results showed that the performance of the NB algorithm was better than that of the emotion lexicon.

### Objectives

Social distancing measures are essential for controlling the spread of infectious diseases during pandemics. However, based on existing research, it is evident that current studies into social distancing have mainly focused on the impact of social distancing measures on the spread of infection rates and mortality. To date, there are few studies that have explored citizens’ emotions related to specific social distancing measures. By detecting the emotional attitudes of Sina Weibo users toward the imposed social distancing measures adopted by Hubei Province, the epicenter of the COVID-19 outbreak in Mainland China, government agencies can take timely action to calm citizens’ emotions. In this study, the following four research questions (RQs) are identified:

- RQ1: what were the emotional attitudes of Sina Weibo users toward the various social distancing measures imposed by Hubei Province?
- RQ2: what were the changing trends in Sina Weibo users’ emotions over time?
- RQ3: what was the impact of user characteristics of social media on their emotions?
- RQ4: what was the moderating effect of social distancing measures on the relationship between the explanatory variables and the explained variable?

## Methods

### Data Collection and Preprocessing

Due to the severity of the COVID-19 outbreak in Hubei Province, the government of Hubei Province issued the “Notice of the People’s Government of Hubei Province on strengthening the prevention and control of the new coronavirus pneumonia” on January 21, 2020 [45], which stipulated strict restrictions against large-scale activities and personal movement; this led to the successful employment of social distancing measures to reduce the risk of infection among citizens. Subsequently, due to improvements in the epidemic situation, the government of Hubei Province announced on March 24, 2020, that it would gradually lift restrictions in the province. Therefore, the time span of the selected data in this study range from January 21 to March 24, 2020.

**Figure 1:**
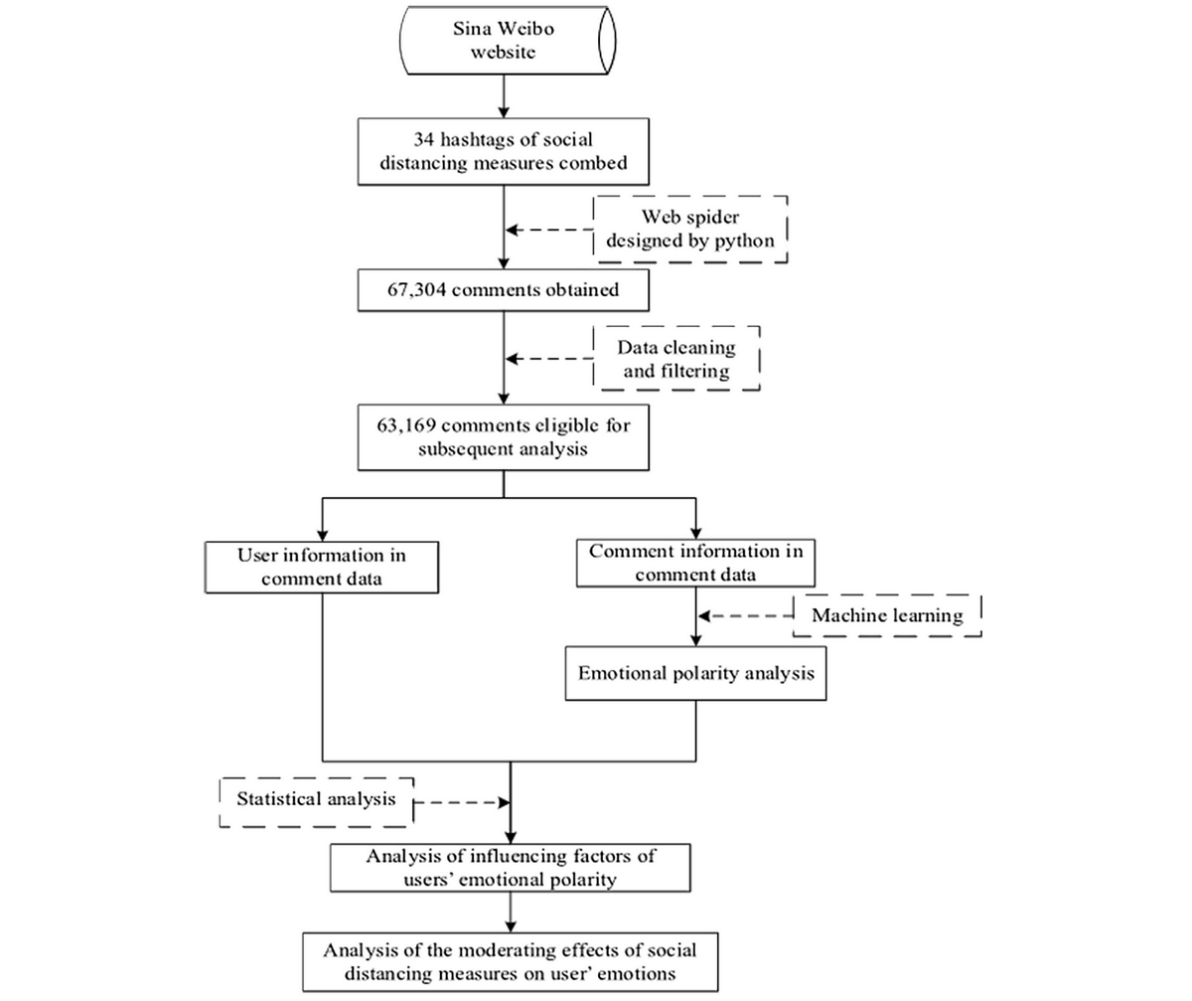
Flowchart for obtaining data from Sina Weibo for subsequent analysis.

In addition, since Sina Weibo has gradually become the main social media platform for Chinese citizens, this study selected this platform as the data source. After combing hashtags on Sina Weibo, which is an efficient method for identifying trending topics [46], a total of 34 hashtags related to social distancing measures adopted by Hubei Province were identified. These hashtags were then categorized under the six social distancing measures, as shown in Table 1. To ensure data quality, comments about posts published by the official news channels of government agencies, certified by the Sina Weibo platform, were crawled using Python 3.7, retrieving a total of 67,304 comments published by 58,996 netizens. After removing duplicate data and comments without textual expression, 63,169 comments were identified. The main data collected included username, gender, spatial location, account registration year, and comment content. The process of subsequent analysis is shown in Figure 1.

**Table 1:** Hashtags on social distancing measures related to COVID-19 on Sina Weibo.

Measures categories	Hashtags
Delaying the resumption of work and school	#The start of the school year for Hubei elementary and middle schools has been postponed# #Colleges and universities in Hubei have postponed the start of classes# #Schools in Hubei have postponed the start of the school year# #Hubei has postponed the start of school# #Hubei continues to delay the resumption of work and school# #The opening of schools in Hubei Province has been postponed# #Hubei continues to delay the start of school# #Hubei issued a notice to continue to delay the resumption of work and schools# #Various enterprises in Hubei will resume work no earlier than 24:00 on February 20# #Enterprises in Hubei Province will resume work no earlier than 24:00 on March 10#
Travel restrictions	#Wuhan canceled all tour groups# #All tour groups in Wuhan will be cancelled# #Hubei travel agencies have suspended business activities# #Hubei province has suspended the operations of travel agencies across the province#
Traffic restrictions	#Wuhan’s public transportation and subway operations have been suspended# #Buses and subways in Wuhan were suspended# #Traffic in Wuhan was suspended# #Long-distance passenger transportation of Wuhan bus, subway and ferry will be suspended from the 23rd# #The Wuhan exit route was temporarily closed# #Huanggang railway station was closed# #Wuhan airport, railway station, and other exit routes were temporarily closed# #Wuhan closed the river-crossing tunnel#
Extending the Lunar New Year holiday	#The Spring Festival holiday was extended to February 2# #Hubei Province will appropriately extend the Spring Festival holiday# #Hubei Province extended the Spring Festival holiday until February 13#
Closing public spaces	#Cinemas throughout Wuhan were temporarily closed# #Wuhan cultural and entertainment venues were temporarily closed# #All star hotel activities in A-level scenic spots in Hubei Province will be cancelled# #All non-essential public places in Hubei were closed#
Community containment	#Communities in Hubei Province are under closed management# #All residential communities in Wuhan are under closed management# #Wuhan community adopted closed management# #The communities in Hubei Province are most strictly closed 24 hours a day# #The closed management of villages and community in the Wuhan will continue 24 hours a day#

### Emotional Polarity Analysis

Since the introduction of text sentiment analysis in 2008, machine learning methods have achieved consistently good results [47,48]. Therefore, we chose machine learning algorithms to perform emotional analysis on the corpus.

First, corpora marking was completed. The current research on emotional analysis mainly divides emotional types into three or five categories [49,50]. In addition, the Ortony-Clore-Collins (OCC) model, proposed by Ortony et al [51] in 1988, is a refined emotion classification model that offers a rule-based emotion export mechanism, which has been widely applied to studies that examine emotion classification of social media users [52]. Based on the three evaluation criteria of the OCC model, consequences of events, action of agents, and aspects of objects, as well as different intensities or inducing causes, this study constructed “anger,” “fear,” “neutral,” “encouragement,” and “hope” emotions to analyze Sina Weibo users’ emotions. The emotions of “anger” and “encouragement” are related to the action of agents, emotions of “fear” and “hope” are related to the consequences of events, and neutral emotions indicate objective facts. Then, according to the above rules of emotion classification, we programed in Python; the first and second authors used traditional labeling methods to label the corpus [53]. We randomly chose more than 5000 corpora and marked them with one of the five emotional polarity. Then, the Kappa coefficient was used to evaluate the consistency of the corpus marking [54]. The Kappa value was 0.95 (*P*<.01), indicating that the marked corpora had strong consistency [55].

We then predicted the emotional polarity of the comments collected. First, we selected four representative training classifiers of machine learning algorithms: support vector machine (SVM), convolutional neural networks (CNN), long short-term memory (LSTM), and bi-directional long short-term memory (Bi-LSTM). Based on the marked corpora, we divided them into training sets, validation sets, and test sets, according to a 6:2:2 ratio, and then input them into different classifier models for testing. These tests were carried out in September 2020. Three indicators—precision, recall, and F1-score—were used for the model evaluation [56]. The precision rate reflects the ability of the classifier to determine the whole sample; the recall rate intuitively reflects the proportion of positive samples that are correctly identified; and the F1-score can be interpreted as a weighted average of precision and recall. As shown in Table 2, the Bi-LSTM classifier exhibited the best performance for testing emotional polarity for the remaining corpora [57]. Subsequently, the Bi-LSTM classifier was used to predict the emotional polarity of all corpora, and this process was performed using Python.

**Table 2:** The performance of the emotional polarity classification model.

Model	Precision	Recall	F1-score
SVM^a^	0.512	0.506	0.509
CNN^b^	0.619	0.602	0.607
LSTM^c^	0.664	0.664	0.658
Bi-LSTM^d^	0.701	0.699	0.701

^a^SVM: support vector machine.

^b^CNN: convolutional neural network.

^c^LSTM: long short-term memory.

^d^Bi-LSTM: bidirectional long short-term memory.

### Research Hypotheses and Statistical Analysis

#### Theoretical Background and Hypotheses Design

To study the emotional attitudes of Sina Weibo users, we examined the impact of user characteristics and social distancing measures on the emotional tendency of users. We then proposed hypotheses.

First, according to the Media Dependency Theory [58], the effects of the media are due to the media meeting the needs of specific audiences in a specific way in a specific society. Obviously, audiences’ use of media platforms determines the media’s influence, namely, personal media dependence depends on personal factors [59]. Therefore, based on the Media Dependence Theory and the data collected on Sina Weibo users, this study hypothesizes that users’ age, spatial location, and social media registration year affects their emotional expression [60,61]. Accordingly, we proposed the following hypotheses:

- H1a: the age of Sina Weibo users has a significant impact on their emotional expression;
- H1b: the spatial location of Sina Weibo users has a significant impact on their emotional expression;
- H1c: the registration year of Sina Weibo users has a significant impact on their emotional expression.

Second, according to the Risk Information Seeking and Processing Model, proposed by Griffin [62], relevant channel beliefs affect individuals’ information processing methods differently [63]. In addition, the 5W Theory (Who, When, Where, What, and How), proposed by Lasswell [64], reveals the communication elements in the communication process. Among them, the first element “who” is played by different types of users on the Sina Weibo platform [65-67]. Therefore, this study measured the emotional response of different user characteristics from the perspective of social media, and proposed the following hypotheses:

- H2a: the number of fans of Sina Weibo users has a significant impact on their emotional expression;
- H2b: the number of follows of Sina Weibo users has a significant impact on their emotional expression;
- H2c: the number of posts shared by Sina Weibo users has a significant impact on their emotional expression.

Third, the Agenda-Setting Theory, formally proposed by McCombs and Shaw [68], posits that mass media can effectively influence users’ attention to certain facts by providing information and arranging related issues. The theory further discusses that records of public discussion on public affairs can be obtained from social media for observation and analysis [69,70]. Based on this theory, we discussed the impact of different social distancing measures on the emotional expression of different types of Sina Weibo users, and proposed the following hypothesis:

- H3: The categories of social distancing measures have a moderating effect on the relationship between the user characteristics of different types of Sina Weibo users and their emotions.

#### Statistical Analysis of Influencing Factors of Emotional Polarity

Based on the above hypotheses, we selected the factors that affect users’ emotions for statistical analysis. Given that the P value of the test of parallel lines is less than .001, we analyzed the factors that influence Sina Weibo users’ emotions using multinomial logistic regression, performed by SPSS 21.0 (IBM Corp); our variables are shown in Table 3. Among the variables, based on the existing research results [71], gender was set as the control variable. Based on related data [72,73], the spatial locations of Sina Weibo users were divided into Hubei Province, emigrant provinces with the largest population influx from Hubei Province before the Wuhan lockdown (including Guangdong, Beijing, Shanghai, Henan, Anhui, Jiangxi, Sichuan, Hunan, and Chongqing), and other provinces. In addition, the effects of various explanatory variables on anger and fear emotions, as well as hope and encouragement emotions, are consistent. Therefore, we merged the five categories of emotions into three categories (positive, neutral, and negative) for statistical analysis. Further, the moderator variables included the six categories of social distancing measures adopted by Hubei Province. In the subsequent analysis, each category was set as the reference group to observe the interaction effect between it and the explanatory variables, which can judge the moderating effect of social distancing measures [74]. From the above, we established the following model:

Logit(P_Emo) = β_0_ + β_1_Sex + β_2_Age + β_3_Space1 + β_4_Space2 + β_5_Ln(N_Fan) + β_6_Ln(N_Follow) + β_7_Ln(N_Post) + β_8_Time_Reg + β_9_T_SDM + β_10_Age × T_SDM + β_11_Space1 × T_SDM + β_12_Space2 × T_SDM + β_13_Ln(N_Fan) × T_SDM + β_14_Ln(N_Follow) × T_SDM + β_15_Ln(N_Post) × T_SDM + β_16_ Time_Reg × T_SDM + ε

**Table 3:** Description of variables that influence Sina Weibo users’ emotions.

Variable		Variable symbol	Description and coding
**Explained variable**			
	Emotional polarity	P_Emo	Sina Weibo users’ emotional polarity
**Control variable**			
	Sex	Sex	The sex of Sina Weibo users. The coding is as follows: 0=female and 1=male (reference group)
**Explanatory variables**			
	Age	Age	The age of Sina Weibo users (range 16-65 years)
	Spatial location	Space1, Space2	The spatial locations of Sina Weibo users. We used “other provinces” as the reference group. The dummy variables are as follows: Space0= {other provinces, when Space1=0 and Space2=0} (reference group) Space1= {1=Hubei Province; 0=others} Space2={1=emigrant provinces; 0=others}
	Number of fans	Ln(N_Fan)	The number of fans of Sina Weibo users; smoothed logarithmically
	Number of follows	Ln(N_Follow)	The number of other users that Sina Weibo users follow; smoothed logarithmically
	Number of post	Ln(N_Post)	The number of posts shared by Sina Weibo users; smoothed logarithmically
	Registration year	Time_Reg	The registration year of Sina Weibo users’ accounts
**Moderator variable**			
	Social distancing measures	T_SDM	The types of social distancing measures. The coding is listed as follows: SDM1=delaying the resumption of work and school SDM2=travel restrictions SDM3= traffic restrictions SDM4=closing public spaces SDM5=community containment SDM6=extending the Lunar New Year holiday (reference group)

In total, 21,395 comments were identified for statistical analysis after removing some records with missing age, since disclosing age is not required for user registration.

## Results

### Basic Description of Comments and Users’ Emotional Polarities

#### Distribution of Comments

**Figure 2:**
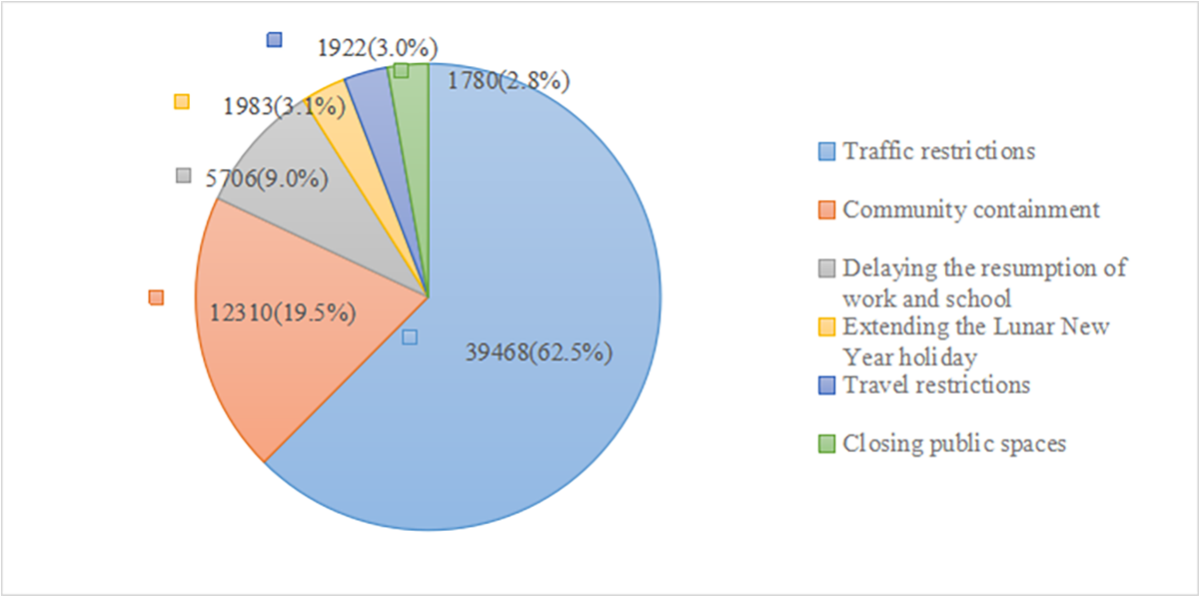
Proportion of comments related to each social distancing measure (N=63,169).

From January 21 to March 24, 2020, Sina Weibo users discussed the social distancing measures imposed by the People’s Government of Hubei Province. The three measures of *traffic restrictions*, *community containment*, and *delaying the resumption of work and school* attracted high attention from users, while *travel restrictions*, *extending the Lunar New Year holiday*, and *closing public places* attracted less attention; further details are shown in Figure 2.

**Figure 3:**
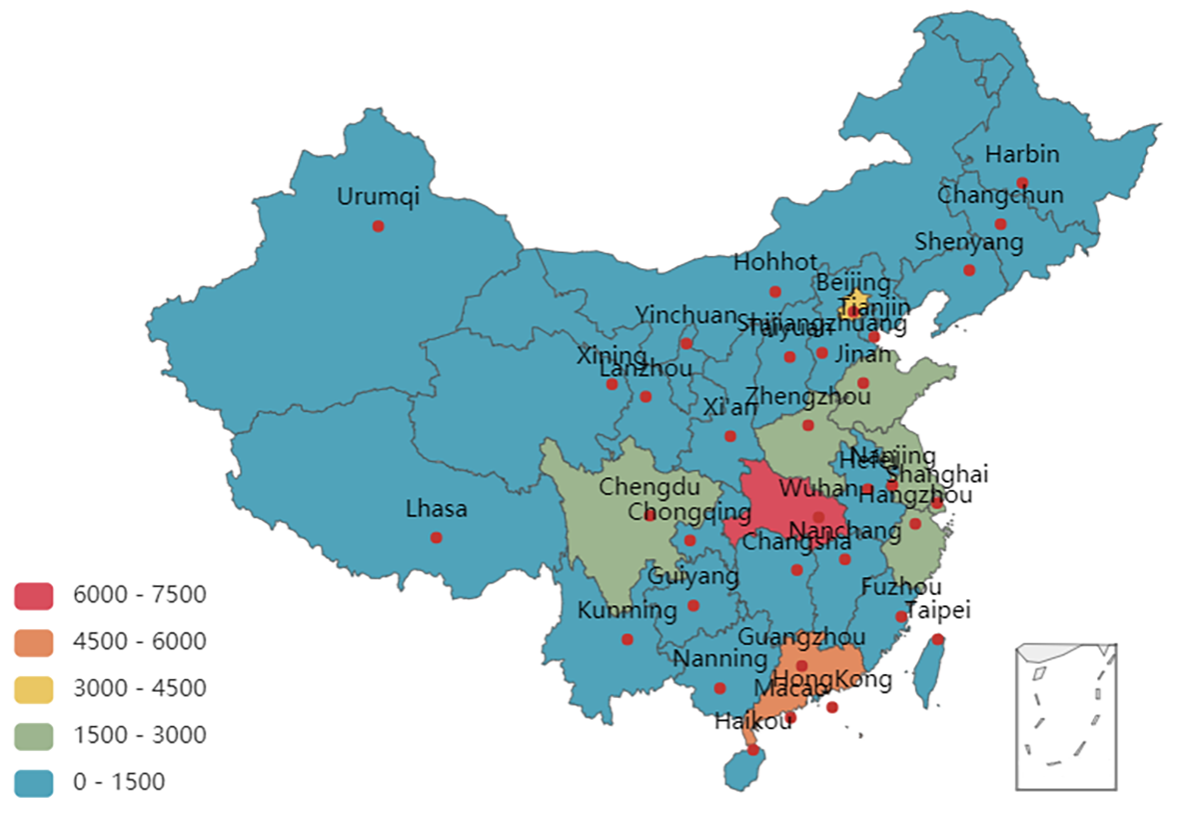
Spatial distribution of comments from Sina Weibo users (N=46,431).

To analyze the data of various provinces in China, the data of users registered as “other” and “overseas” were removed, yielding 46,431 comments. Local netizens in Hubei Province paid the greatest attention to social distancing measures, followed by those residing in the Guangdong, Beijing, and Shanghai. These are areas where citizens from Hubei Province relocated to before the lockdown was imposed in Wuhan City. The number of comments from users in other provinces was relatively small, as shown in Figure 3.

#### Distribution of Users’ Emotional Polarity Across Gender

The findings show that women held a higher proportion of positive emotions than men (Table 4). Further, the chi-square test result (*χ*^2^_4_=1784.59, *P*<.001) shows that the emotional expression of different genders varied.

**Table 4:** Proportion of users’ emotional polarities across different genders (N=63,169).

Sex	Positive		Neutral	Negative	
	Hope	Encouragement		Fear	Anger
Male (n=19,016), n (%)	1512 (8.0)	4505 (23.7)	2896 (15.2)	4467 (23.5)	5636 (29.6)
Female (n=44,153), n (%)	4383 (9.9)	16,936 (38.4)	4207 (9.5)	9601 (21.7)	9026 (20.4)

#### Distribution of Emotional Polarity Among Users From Different Spatial Locations

**Figure 4:**
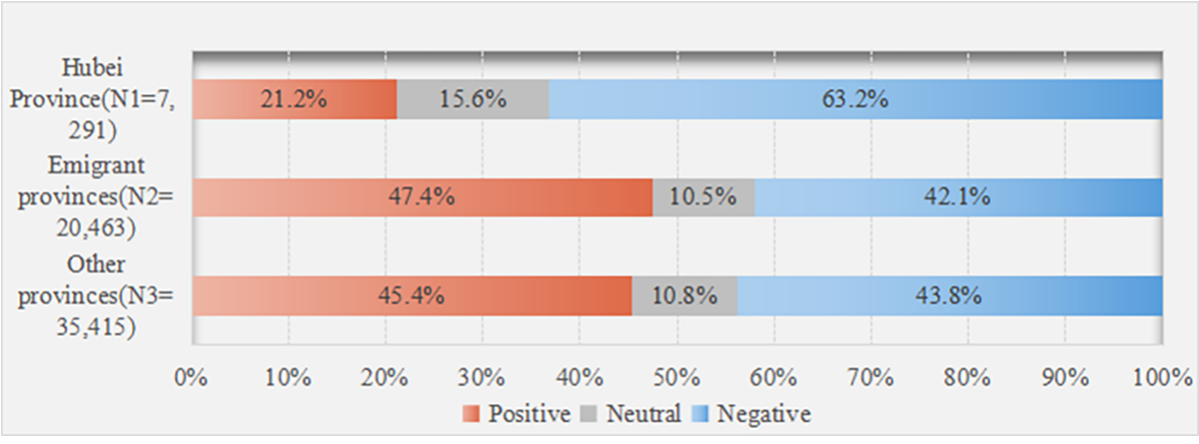
Proportion of users' emotional polarity from different spatial locations (N=63,169).

As shown in Figure 4, for users in Hubei Province, the proportion of negative emotions (4605/7291, 63.2%) is significantly different from that of positive emotions (1545/7291, 21.2%). Sina Weibo users in Hubei Province showed the least amount of positive emotions while residents of emigrant provinces expressed the most positive emotions. There was little difference between the emigrant provinces and other provinces, but the proportion of negative emotions was slightly lower than that of other provinces. In addition, the chi-square test result (*χ*^2^_4_=1659.67, *P*<.001) shows that the emotional expression of users in different spatial locations is indeed different.

### Evolution of Emotional Polarity

#### Distribution of Users’ Emotional Polarity Based on Different Social Distancing Measures

**Figure 5:**
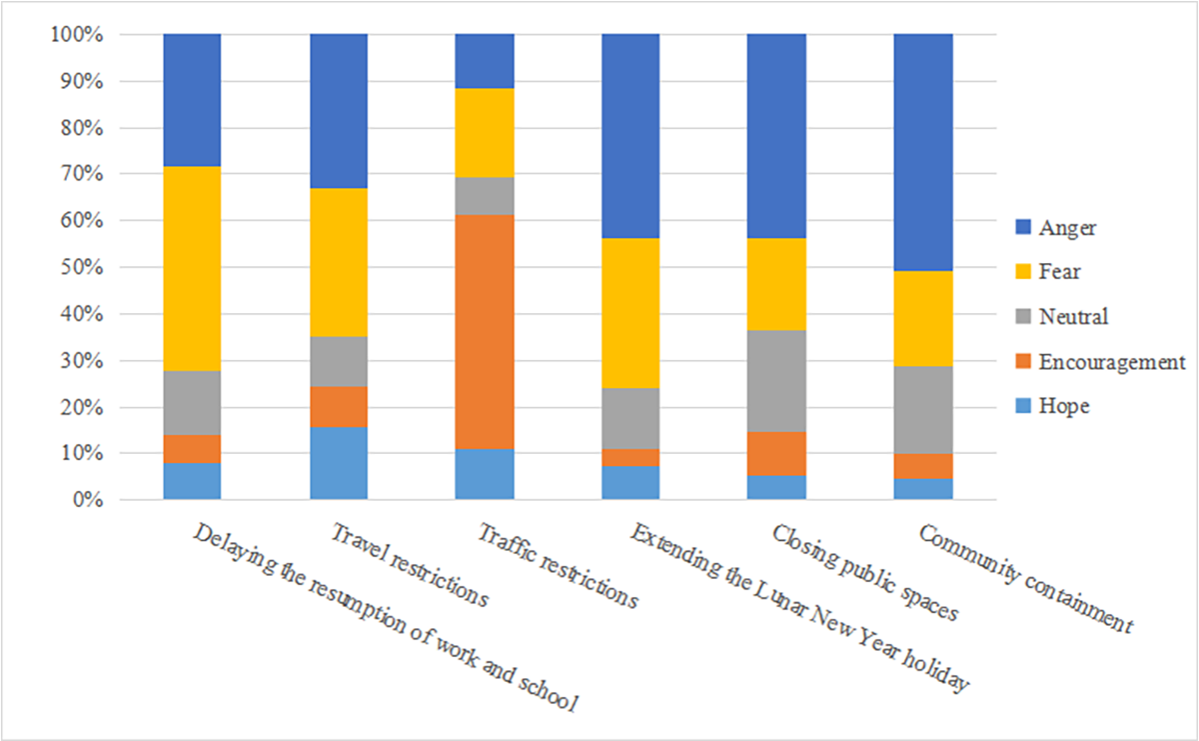
Distribution of users' emotional polarity under various social distancing measures (N=63,169).

Except for the measure of *traffic restrictions*, netizens’ emotions regarding the other five measures were all negatively inclined (Figure 5). Specifically, for the measure of *traffic restrictions*, netizens’ positive emotions make up the largest proportion (24,143/39,468, 61.2%) among all the measures. For *delaying the resumption of work and schoo*l, netizens mostly expressed negative emotions (4129/5706, 72.4%), such as fear and anger. For *travel restrictions*, emotions surrounding hope accounted for the highest proportion (472/1922, 15.6%). In terms of *extending the Lunar New Year holiday*, *closing public spaces*, and *community containment*, negative emotions accounted for a high proportion of total comments. For *community containment*, anger was expressed more frequently compared to all other measures. The chi-square test result (*χ*^2^_20_=19,084.73, *P*<.001) showed that Sina Weibo users have varying emotional expressions for different topics.

#### Evolution Process of Users’ Emotional Polarity Over Time

**Figure 6:**
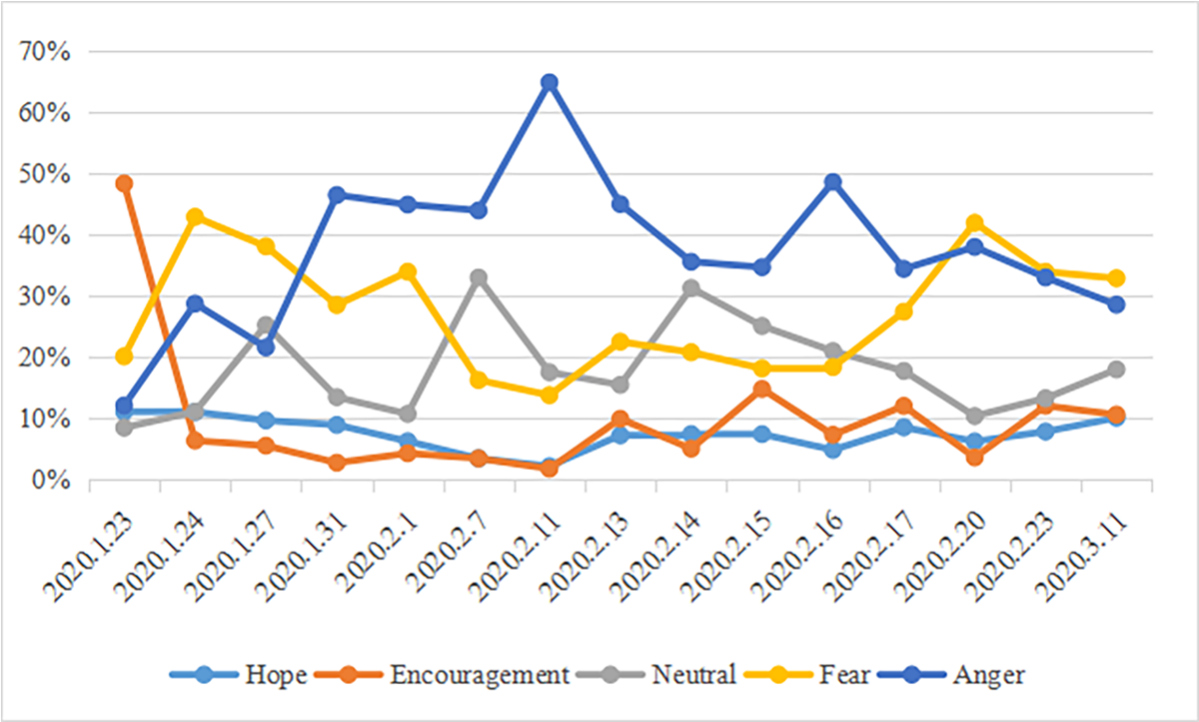
Evolution process of emotional polarity over time (N=63,169).

As shown in Figure 6, on January 23, 2020, when the city of Wuhan was locked down, users showed a high degree of encouragement. However, since January 24, 2020, negative emotions gradually increased with users expressing fear and anger toward the imposed social distancing measures employed by Hubei Province. Then, after January 27, 2020, users’ negative emotions centered on anger toward the social distancing measures imposed in Hubei Province.

### Moderating Effects of Social Distancing Measures on the Influencing Factors of Emotional Polarity

The influence of explanatory variables on the explained variable is shown in Table 5. The reference group of emotional polarity is neutral emotions, and there was no multicollinearity in the data. Older citizens were more inclined to express neutral attitudes. Users in Hubei Province were more likely to express negative emotions, although there was no statistically significant difference in emotional tendency between emigrant provinces and other provinces. Further, users with accounts registered for longer periods of time were inclined to express negative emotions. Similarly, users with more follows and posts were inclined to express positive attitudes, while users with more fans were inclined to express neutral attitudes. Therefore, the explanatory variables do have an impact on the explained variable. Accordingly, hypotheses H1a-c and H2a-c were supported.

**Table 5:** Analysis of the influence of explanatory variables on explained variables (N=21,395).

Variables	Negative, OR^a^ (95% CI)	Positive, OR (95% CI)
Sex (reference group: male)	1.18 (1.05-1.32^b^)	2.21 (1.96-2.49^b^)
Age	0.99 (0.98-0.99^b^)	0.97 (0.98-0.98^b^)
Space0 (reference group)	—^c^	—
Space1	1.18 (1.02-1.38^b^)	0.52 (0.44-0.62^b^)
Space2	1.09 (0.97-1.22)	1.10 (0.98-1.24)
Time_Reg^d^	1.02 (0.99-1.04)	0.90 (0.88-0.93^b^)
Ln(N_Fan)^e^	0.97 (0.94-1.01)	0.98 (0.94-0.99^b^)
Ln(N_Follow)^f^	1.01 (1.02-1.08^b^)	1.05 (0.99-1.12)
Ln(N_Post)^g^	0.96 (0.93-0.99^b^)	1.06 (1.03-1.10^b^)
SDM1^h^	0.49 (0.31-0.78^b^)	7.66 (4.99-11.77^b^)
SDM2^i^	0.46 (0.28-0.75^b^)	6.75 (4.14-11.00^b^)
SDM3^j^	0.59 (0.38-0.92^b^)	1.08 (0.73-1.59)
SDM4^k^	0.92 (0.46-1.81)	0.70 (0.38-1.28)
SDM5^l^	0.56 (0.36-0.88^b^)	8.63 (5.68-13.11^b^)
SDM6^m^ (reference group)	—	—

^a^OR: odds ratio.

^b^*P*<.05.

^c^Not applicable.

^d^Time_Reg: registration year.

^e^Ln(N_Fan): logarithm of fan numbers.

^f^Ln(N_Follow): logarithm of follow numbers.

^g^Ln(N_Post): logarithm of post numbers.

^h^SDM1: delaying the resumption of work and school.

^i^SDM2: travel restrictions.

^j^SDM3: traffic restrictions.

^k^SDM4: closing public spaces.

^l^SDM5: community containment.

^m^SDM6: extending the Lunar New Year holiday.

To examine the effects of the moderating variable on the relationship between users’ characteristics and emotions (positive or negative), interactions between social distancing measures and explanatory variables were observed through binary logistic regression analysis. During this process, 64 outliers were removed, based on the residuals analysis, making the results more meaningful. The omnibus tests of model coefficients were statistically significant (*χ*^2^_48_=4994.56, *P*<.001), and the Hosmer-Lemeshow goodness-of-fit was 0.24 (*χ*^2^_8_=10.34, *P*>.24), indicating that our model has a good goodness of fit. In review of the interaction term results for each social distancing measure of the moderating variable and the explanatory variable, including the odds ratio and 95% CI, it was found that compared with other social distancing measures, some measures had a positive or negative emotion regulation effect on the relationship between the explanatory variable and emotional attitude.

Table 6 summarizes the statistically significant emotional tendency of each explanatory variable under each social distancing measure (for the details of interaction effect, see Multimedia Appendix 1). In general, for these explanatory variables, compared with other types of social distancing measures, the measures of delaying the resumption of work and school (SDM1) or travel restrictions (SDM2) mainly had a positive moderating effect on public emotion, while the measures of traffic restrictions (SDM3) or community containment (SDM5) mainly had a negative moderating effect on public emotion. For the explanatory variable Ln(N_Post), the emotional regulation effects of SDM3 and SDM5 were positive. Different social distancing measures had differing moderating effects on the relationship between user characteristics and user emotions. Therefore, hypothesis H3 is valid.

**Table 6:** Statistics on the emotional tendency of explanatory variables under the moderating effect of various social measures.

Variable	The measures make the explanatory variables have a positive tendency toward emotions	The measures make the explanatory variables have a negative tendency toward emotions
Age	SDM1^a^, SDM2^b^, SDM3^c^	SDM4^d^, SDM5^e^, SDM6^f^
Space1	SDM1, SDM2	SDM3, SDM4, SDM5
Time_Reg^g^	SDM1, SDM2, SDM3, SDM6	SDM5
Ln(N_Fan)^h^	SDM2	SDM1, SDM3
Ln(N_Follow)^i^	SDM1	SDM2, SDM3
Ln(N_Post)^j^	SDM1, SDM3, SDM5	SDM2, SDM6

^a^SDM1: delaying the resumption of work and school.

^b^SDM2: travel restrictions.

^c^SDM3: traffic restrictions.

^d^SDM4: closing public spaces.

^e^SDM5: community containment.

^f^SDM6: extending the Lunar New Year holiday.

^g^Time_Reg: registration year.

^h^Ln(N_Fan): logarithm of fan numbers.

^i^Ln(N_Follow): logarithm of follow numbers.

^j^Ln(N_Post): logarithm of post numbers.

## Discussion

In this study, the comments shared by Sina Weibo users related to the social distancing measures imposed by the People’s Government of Hubei Province during the COVID-19 pandemic were examined. Machine learning and statistical analysis were used to reveal the emotional attitudes of users toward social distancing measures, as well as the effect of different user characteristics and social distancing measures on the users’ emotions, especially the moderating effect of social distancing measures on the relationship between user characteristics and users’ emotions.

### Sina Weibo Users’ Attention to Social Distancing Measures Varied

Sina Weibo users paid varying attention to the social distancing measures imposed by the People’s Government of Hubei Province. The three measures of *traffic restrictions*, *community containment*, and *delaying resumption of work and school* attracted public attention. The main reason for this is that these measures are closely related to the transportation and daily lives of citizens in Hubei Province. When the epidemic situation became more serious, citizens paid greater attention to the daily travel of medical staff and the guaranteeing of living materials for citizens in quarantine. Although the measures of *extending the Lunar New Year holiday*, *travel restrictions*, and *closing public places* are necessary for epidemic prevention and control, they are less relevant to the daily lives of citizens living in Hubei Province. Therefore, these measures were less concerning for users.

In addition, Sina Weibo users in different spatial locations expressed differing levels of attention to social distancing measures imposed in Hubei Province. Users residing in Hubei Province are at the center of the epidemic and felt more deeply about the relevant measures. They are also the group most concerned about social distancing measures. Secondly, for Sina Weibo users in emigrant provinces, such as Guangdong and Beijing, where people in Hubei Province went after the lockdown of Wuhan, these people mainly included students and workers who went to school and work in Hubei Province and, therefore, became worried about the safety of their long-term residence. In particular, Guangdong confirmed the first case of the coronavirus on January 19, 2020, becoming the first province in Mainland China to have a confirmed case of COVID-19 outside of Hubei Province [75]. Sina Weibo users in other provinces paid less attention to Hubei Province since they reside far away from the region. Further, results of our emotional polarity analyses show that users from Hubei Province expressed strong negative emotions, while users in emigrant and other provinces expressed stronger positive emotions, indicating that people at the center of the outbreak have strong negative emotions [61]. As for gender, women shared stronger and more positive emotions than men [71].

### Sina Weibo Users’ Attitudes Toward Social Distancing Measures Were Mainly Negative

The emotional polarity analysis revealed the attitudes of Sina Weibo users toward the social distancing measures imposed in Hubei Province. The emotional analysis results of the whole corpus showed that the emotions of users were mainly negative, which indicated that although social distancing measures had brought great benefits to the prevention and control of the epidemic in Hubei Province, they still inevitably affected citizens’ lives in a negative way [76]. As for the social distancing measures imposed, the measure of *traffic restrictions* was promulgated at the initial stage of the epidemic, together with the notice of the Wuhan lockdown, with users residing outside of Hubei Province supporting and encouraging this measure. However, after the measure of *extending the Lunar New Year holiday* was imposed on January 27, 2020, citizens gradually realized the threat to their lives brought on by COVID-19. At the same time, a series of measures had been imposed to disturb citizens daily lives and, therefore, the negative emotions of fear and anger remained high. For example, under the measures of *delaying the resumption of work and school* and *extending the Lunar New Year holiday*, citizens began to worry about their studies, job security, and future family income [77]. After the traffic ban was imposed, people worried about travel outside of their homes, especially medical staff. For *community containment*, people were forced to stay at home, resulting in a reduction in life satisfaction, which led to an increased feeling of loneliness and psychological distress. Further, loneliness, which is likely to be exacerbated through greater fear and confusion, was seen to increase the risk of mental and physical diseases [78]. For this, more remote assistance, such as psychological counseling for families, was needed.

### Sina Weibo Users With Different Social Media Characteristics Have Varying Emotional Tendencies

The results of the logistic regression analysis supported the results of our emotional polarities analysis. Women were indeed more likely to express stronger and more positive emotions. Users in Hubei Province, who were at the center of the epidemic, were more inclined to express negative emotions. For users of different ages, older citizens were inclined to send neutral comments, possibly because younger users do not have enough life experiences to distinguish right from wrong and tend to accept all information without question. This is consistent with the research of Holmes [79], who found that the COVID-19 pandemic had a significant negative impact on social groups, especially young people. In addition, users who had been using Sina Weibo for a longer period of time were more likely to share negative comments, possibly because they have a higher risk perception than those who seldom use social media [80]. Further, users with more fans and follows and those who shared a greater number of posts were inclined to express neutral or positive attitudes. For this result, we understand that users with ample follows can obtain information from multiple sources to identify relatively positive information and disseminate it. Similarly, users with a large number of fans are considered to be somewhat influential in their community [81] and hold an attitude of being responsible to their fans and, therefore, tend to post objective and positive comments to prevent fans from panicking due to excessive processing of information. Therefore, users with many fans can play a guiding role by posting objective facts or words of encouragement, so as to reduce the negative emotions experienced by fans. Social media providers should also give full attention to the role that their platform plays in the lives of citizens, providing comprehensive and accurate information to citizens [82]. They should also send appropriate notifications to prevent negative mental health.

### Attention Should Be Paid to the Moderating Effect of Social Distancing Measures

The moderating effect of social distancing measures on the relationship between different user characteristics and emotions of users varied. In public health emergencies, for all six social distancing measures, the measures of *delaying the resumption of work and school* or *travel restrictions* may be met with more acceptance by citizens. *Travel restrictions* during the epidemic are an inevitable measure, especially since the epidemic occurred during the Lunar New Year holiday. At the time, many residents left Wuhan to travel to see relatives, making the spread of the disease a more serious issue [83]. Tourism is not very important in the face of personal safety, so people are more accepting of travel restrictions. Further, the measures of *traffic restrictions* or *community containment* are easily accepted by citizens. The emotion results show that these measures were associated with the most positive emotions; this is because when these measures were first promulgated, people believed their implementation could cut off routes of viral transmission and be effective for epidemic prevention and control. However, continuous traffic restrictions have brought significant inconvenience to citizens’ travel plans, especially those related to employment. In response to this situation, government departments should take timely measures to resolve this problem, such as arranging specialized personnel or calling on more volunteers to provide convenience to those who need to travel, so as to alleviate the negative emotions of people. In particular, the measure of *community containment* is shown to increase health anxiety, financial worry, and loneliness [84,85], and may lead to depression and anxiety among older citizens. For this, governments must take action to strengthen interactions within local communities [86] and provide remote assistance, such as counseling for families, to reduce the psychological burden of citizens.

### Implications and Limitations

This study has a good degree of theoretical value. We explored the relevant characteristics that affect users’ emotions from the perspective of the Media Dependence Theory, 5W Theory, and Agenda-Setting Theory, and then analyzed the influence relationship between user characteristics and users’ emotions. This study has enriched the research directions of these three theories from a new perspective and has created a certain reference value for future studies.

In addition, our results have a degree of practical significance. We found that Sina Weibo users’ views and attitudes toward social distancing measures imposed by the People’s Government of Hubei Province varied. Users with different characteristics also had different emotional tendencies. In particular, social distancing measures had a moderating effect on the relationship between user characteristics and users’ emotions. These results are helpful for government agencies to uncover, in a timely manner, citizens’ emotions pertaining to measure implementations. Our findings also provide guidelines for social media platforms to push targeted content to users.

This study has several limitations. First, due to restrictions by Sina Weibo, the secondary comment data below the posts was not obtained, which may affect the results and discussion presented. Second, the training data labels of emotion classifiers were mainly marked manually through the establishment of labeling guidelines. Further, the expression of emotion is highly subjective, and manual labeling may not reflect the real emotions of users adequately. These factors may affect the classification effect of emotional polarity classifiers to a certain extent. Finally, this study only analyzed the emotions in the text without considering emoticons, which may have a certain influence on the results of emotion classification. Future research should consider the effect of emoticons and punctuation on emotional intensity.

### Conclusions

This study combined machine learning and statistical analysis to explore the emotional attitudes of citizens toward social distancing measures imposed by the People’s Government of Hubei Province, as well as these emotions’ influencing factors. The results of our emotional analysis show that Sina Weibo users have different attitudes toward the six types of social distancing measures implemented, but they are mainly inclined to express negative emotions. In addition, users’ emotional attitudes vary across gender and spatial locations. The logistic regression analysis show that users of different ages, spatial locations, account registration year, number of fans, number of follows, and number of posts have different attitudes toward the imposed social distancing measures. Most importantly, this study found that social distancing measures have a moderating effect on the relationship between different user characteristics and users’ emotions. The results obtained allow government agencies to better understand the views of citizens toward related events and can help government agencies take timely actions to alleviate negative emotions during public health emergencies.

## Appendix

Multimedia Appendix 1 The interaction effect of the social distancing measures and explanatory variables.

## Abbreviations

Bi-LSTM: bi-directional long short-term
CNN: convolutional neural network
LSTM: long short-term memory
NB: naive Bayes
NRC: National Research Council of Canada
OCC: Ortony-Clore-Collins model
RQ: research question
SARS: severe acute respiratory syndrome
SVM: support vector machine
