# Supplementary material for: Emotional Attitudes of Chinese Citizens on Social Distancing During the COVID-19 Outbreak: Analysis of Social Media Data
Source: JMIR Med Inform. 2021 Mar 16;9(3):e27079. doi: 10.2196/27079 (PMC7968412; doi:10.2196/27079)
Supplement: Multimedia Appendix 1 [file medinform_v9i3e27079_app1.doc]

# Appendix

Table A: The details of interaction effect of the social distancing measures and explanatory variables (N=16,194).

|  | Reference category | Positive-Negative | | Reference category | Positive-Negative | | Reference category | Positive-Negative | |
| --- | --- | --- | --- | --- | --- | --- | --- | --- | --- |
|  | SDM1^a^ | *β*^b^ | OR^c^ (95%CI^d^) | SDM2^e^ | *β* | OR (95%CI) | SDM3^f^ | *β* | OR (95%CI) |
| Age× | SDM2 | 0.02 | 1.02(0.98-1.06) | SDM1 | -0.02 | 0.98(0.95-1.02) | SDM1 | 0.05 | 1.06(1.03-1.08)^g^ |
|  | SDM3 | -0.05 | 0.95(0.93-0.97)^g^ | SDM3 | -0.07 | 0.93(0.90-0.96)^g^ | SDM2 | 0.07 | 1.07(1.04-1.11)^g^ |
|  | SDM4^h^ | -0.78 | 0.46(023-0.93)^g^ | SDM4 | -0.80 | 0.45(0.22-0.92)^g^ | SDM4 | -0.73 | 0.48(0.24-0.98)^g^ |
|  | SDM5^i^ | -0.11 | 0.90(0.87-0.92)^g^ | SDM5 | -0.13 | 0.88(0.85-0.91)^g^ | SDM5 | -0.06 | 0.95(0.93-0.96)^g^ |
|  | SDM6^j^ | -0.99 | 0.37(0.17-0.83)^g^ | SDM6 | -1.01 | 0.36(0.16-0.81)^g^ | SDM6 | -0.94 | 0.39(0.18-0.87)^g^ |
| Space1× | SDM2 | 0.46 | 1.59(0.74-3.42) | SDM1 | -0.46 | 0.63(0.29-1.36) | SDM1 | 0.45 | 1.58(1.04-2.38)^g^ |
|  | SDM3 | -0.45 | 0.64(0.42-0.96)^g^ | SDM3 | -0.92 | 0.40(0.20-0.79)^g^ | SDM2 | 0.92 | 2.50(1.27-4.91)^g^ |
|  | SDM4 | -1.66 | 0.19(0.06-0.61)^g^ | SDM4 | -2.12 | 0.12(0.03-0.43)^g^ | SDM4 | -1.20 | 0.30(0.10-0.90)^g^ |
|  | SDM5 | -0.42 | 0.66(0.37-1.18) | SDM5 | -0.89 | 0.41(0.19-0.91)^g^ | SDM5 | 0.03 | 1.03(0.65-1.63) |
|  | SDM6 | -0.75 | 0.48(0.20-1.15) | SDM6 | -1.21 | 0.30(0.11-0.84)^g^ | SDM6 | -0.29 | 0.75(0.33-1.68) |
| Space2× | SDM2 | 0.10 | 1.10(0.61-2.01) | SDM1 | -0.10 | 0.91(0.50-1.65) | SDM1 | 0.08 | 1.08(0.76-1.54) |
|  | SDM3 | -0.08 | 0.92(0.65-1.32) | SDM3 | -0.18 | 0.84(0.51-1.38) | SDM2 | 0.18 | 1.19(0.73-1.97) |
|  | SDM4 | -0.52 | 0.59(0.27-1.30) | SDM4 | -0.62 | 0.54(0.23-1.27) | SDM4 | -0.44 | 0.64(0.32-1.31) |
|  | SDM5 | -0.21 | 0.81(0.52-1.27) | SDM5 | -0.31 | 0.74(0.42-1.30) | SDM5 | -0.13 | 0.88(0.66-1.18) |
|  | SDM6 | 0.20 | 1.22(0.65-2.31) | SDM6 | 0.10 | 1.11(0.54-2.29) | SDM6 | 0.28 | 1.33(0.77-2.28) |
| Time_Reg^k^× | SDM2 | 0.05 | 1.05(0.94-1.17) | SDM1 | -0.05 | 0.96(0.86-1.07) | SDM1 | 0.01 | 1.01(0.94-1.07) |
|  | SDM3 | -0.01 | 1.00(0.93-1.06) | SDM3 | -0.05 | 0.95(0.87-1.05) | SDM2 | 0.05 | 1.05(0.96-1.16) |
|  | SDM4 | -0.17 | 0.85(0.69-1.04) | SDM4 | -0.21 | 0.81(0.65-1.01) | SDM4 | -0.16 | 0.85(0.70-1.04) |
|  | SDM5 | -0.13 | 0.88(0.81-0.96)^g^ | SDM5 | -0.17 | 0.84(0.76-0.94)^g^ | SDM5 | -0.12 | 0.89(0.84-0.94)^g^ |
|  | SDM6 | 0.05 | 1.05(0.91-1.22) | SDM6 | 0.01 | 1.01(0.86-1.19) | SDM6 | 0.06 | 1.06(0.93-1.21) |
| Ln(N_Fan)^l^× | SDM2 | 0.19 | 1.21(1.02-1.44)^g^ | SDM1 | -0.19 | 0.83(0.70-0.98)^g^ | SDM1 | -0.03 | 0.97(0.88-1.08) |
|  | SDM3 | 0.03 | 1.03(0.93-1.14) | SDM3 | -0.16 | 0.85(0.74-0.98)^g^ | SDM2 | 0.16 | 1.18(1.02-1.36)^g^ |
|  | SDM4 | 0.03 | 1.03(0.77-1.36) | SDM4 | -0.16 | 0.85(0.63-1.14) | SDM4 | 0 | 1.00(0.77-1.30) |
|  | SDM5 | 0.10 | 1.11(0.98-1.26) | SDM5 | -0.09 | 0.92(0.78-1.08) | SDM5 | 0.08 | 1.08(0.99-1.17) |
|  | SDM6 | 0 | 1.00(0.81-1.24) | SDM6 | -0.19 | 0.83(0.65-1.05) | SDM6 | -0.03 | 0.97(0.80-1.18) |
| Ln(N_  Follow)^m^× | SDM2 | -0.34 | 0.72(0.53-0.97)^g^ | SDM1 | 0.34 | 1.40(1.03-1.90)^g^ | SDM1 | 0.2 | 1.22(1.02-1.47)^g^ |
|  | SDM3 | -0.20 | 0.82(0.68-0.98)^g^ | SDM3 | 0.14 | 1.15(0.89-1.48) | SDM2 | -0.14 | 0.87(0.68-1.13) |
|  | SDM4 | -0.17 | 0.85(0.55-1.30) | SDM4 | 0.17 | 1.19(0.75-1.88) | SDM4 | 0.03 | 1.04(0.70-1.53) |
|  | SDM5 | -0.22 | 0.81(0.64-1.01) | SDM5 | 0.12 | 1.13(0.84-1.51) | SDM5 | -0.02 | 0.98(0.84-1.15) |
|  | SDM6 | -0.29 | 0.75(0.53-1.06) | SDM6 | 0.04 | 1.05(0.70-1.55) | SDM6 | -0.09 | 0.91(0.67-1.25) |
| Ln(N_Post)^n^× | SDM2 | -0.08 | 0.92(0.79-1.08) | SDM1 | 0.08 | 1.08(0.92-1.27) | SDM1 | -0.08 | 0.93(0.84-1.02) |
|  | SDM3 | 0.08 | 1.08(0.98-1.19) | SDM3 | 0.16 | 1.17(1.02-1.34)^g^ | SDM2 | -0.16 | 0.86(0.75-0.98)^g^ |
|  | SDM4 | 0.15 | 1.16(0.94-1.44) | SDM4 | 0.23 | 1.26(1.00-1.59)^g^ | SDM4 | 0.07 | 1.08(0.89-1.30) |
|  | SDM5 | 0.13 | 1.14(1.01-1.29)^g^ | SDM5 | 0.21 | 1.24(1.06-1.44)^g^ | SDM5 | 0.06 | 1.06(0.97-1.15) |
|  | SDM6 | -0.07 | 0.93(0.79-1.10) | SDM6 | 0.01 | 1.01(0.83-1.22) | SDM6 | -0.15 | 0.86(0.75-0.99)^g^ |
| Constant | -2.97 | | | -2.31 | | | 1.36 | | |
| ^a^SDM1: delaying the resumption of work and school  ^b^*β*: Beta coefficient  ^c^OR: odds ratio  ^d^95% CI:: 95% confidence interval  ^e^SDM2: travel restrictions  ^f^SDM3: traffic restrictions  ^g^ *P*<.05  ^h^SDM4: closing public spaces  ^i^SDM5: community containment  ^j^SDM6: extending the Lunar New Year holiday  ^k^Time_Reg: Registration year  ^l^Ln(N_Fan): Logarithm of fan numbers  ^m^Ln(N_Follow): Logarithm of follow numbers  ^n^Ln(N_Post): Logarithm of post numbers | | | | | | | | | |

Continued Table A.

|  | Reference category | Positive-Negative | | Reference category | Positive-Negative | | Reference category | Positive-Negative | |
| --- | --- | --- | --- | --- | --- | --- | --- | --- | --- |
|  | SDM4 | *β* | OR (95%CI) | SDM5 | *β* | OR (95%CI) | SDM6 | *β* | OR (95%CI) |
| Age× | SDM1 | 0.78 | 2.19(1.07-4.45)^g^ | SDM1 | 0.11 | 1.12(1.09-1.15)^g^ | SDM1 | 0.99 | 2.70(1.21-6.01)^g^ |
|  | SDM2 | 0.8 | 2.22(1.09-4.53)^g^ | SDM2 | 0.13 | 1.14(1.10-1.18)^g^ | SDM2 | 1.01 | 2.75(1.23-6.12)^g^ |
|  | SDM3 | 0.73 | 2.07(1.02-4.21)^g^ | SDM3 | 0.06 | 1.06(1.04-1.08)^g^ | SDM3 | 0.94 | 2.56(1.15-5.70)^g^ |
|  | SDM5 | 0.67 | 1.96(0.96-3.99) | SDM4 | -0.67 | 0.51(0.25-1.04) | SDM4 | 0.21 | 1.23(0.42-3.60) |
|  | SDM6 | -0.21 | 0.81(0.28-2.36) | SDM6 | -0.88 | 0.41(0.19-0.92)^g^ | SDM5 | 0.88 | 2.42(1.08-5.39)^g^ |
| Space1× | SDM1 | 1.66 | 5.23(1.65-16.54)^g^ | SDM1 | 0.42 | 1.53(0.85-2.74) | SDM1 | 0.75 | 2.11(0.87-5.11) |
|  | SDM2 | 2.12 | 8.30(2.33-29.52)^g^ | SDM2 | 0.89 | 2.42(1.10-5.35)^g^ | SDM2 | 1.21 | 3.34(1.19-9.41)^g^ |
|  | SDM3 | 1.20 | 3.32(1.12-9.89)^g^ | SDM3 | -0.03 | 0.97(0.62-1.53) | SDM3 | 0.29 | 1.34(0.60-2.99) |
|  | SDM5 | 1.23 | 3.43(1.07-11.00)^g^ | SDM4 | -1.23 | 0.29(0.09-0.94)^g^ | SDM4 | -0.91 | 0.40(0.11-1.54) |
|  | SDM6 | 0.91 | 2.48(0.65-9.52) | SDM6 | -0.32 | 0.73(0.29-1.80) | SDM5 | 0.32 | 1.38(0.56-3.42) |
| Space2× | SDM1 | 0.52 | 1.69(0.77-3.70) | SDM1 | 0.21 | 1.23(0.79-1.92) | SDM1 | -0.20 | 0.82(0.43-1.54) |
|  | SDM2 | 0.62 | 1.86(0.79-4.40) | SDM2 | 0.31 | 1.36(0.77-2.39) | SDM2 | -0.10 | 0.90(0.44-1.86) |
|  | SDM3 | 0.44 | 1.56(0.77-3.18) | SDM3 | 0.13 | 1.14(0.85-1.53) | SDM3 | -0.28 | 0.76(0.44-1.30) |
|  | SDM5 | 0.32 | 1.37(0.64-2.93) | SDM4 | -0.32 | 0.73(0.34-1.56) | SDM4 | -0.73 | 0.48(0.20-1.17) |
|  | SDM6 | 0.73 | 2.07(0.85-5.00) | SDM6 | 0.41 | 1.51(0.82-2.75) | SDM5 | -0.41 | 0.66(0.36-1.21) |
| Time_Reg× | SDM1 | 0.17 | 1.18(0.96-1.46) | SDM1 | 0.13 | 1.13(1.05-1.23)^g^ | SDM1 | -0.05 | 0.95(0.82-1.10) |
|  | SDM2 | 0.21 | 1.24(0.99-1.54) | SDM2 | 0.17 | 1.19(1.07-1.32)^g^ | SDM2 | -0.01 | 0.99(0.84-1.17) |
|  | SDM3 | 0.16 | 1.18(0.96-1.44) | SDM3 | 0.12 | 1.13(1.07-1.19)^g^ | SDM3 | -0.06 | 0.94(0.83-1.08) |
|  | SDM5 | 0.04 | 1.04(0.85-1.28) | SDM4 | -0.04 | 0.96(0.78-1.18) | SDM4 | -0.22 | 0.80(0.63-1.02) |
|  | SDM6 | 0.22 | 1.25(0.98-1.58) | SDM6 | 0.18 | 1.20(1.04-1.38)^g^ | SDM5 | -0.18 | 0.84(0.73-0.96)^g^ |
| Ln(N_Fan)× | SDM1 | -0.03 | 0.98(0.74-1.29) | SDM1 | -0.10 | 0.90(0.80-1.02) | SDM1 | 0 | 1.00(0.81-1.24) |
|  | SDM2 | 0.16 | 1.18(0.87-1.59) | SDM2 | 0.09 | 1.09(0.93-1.28) | SDM2 | 0.2 | 1.21(0.95-1.53) |
|  | SDM3 | 0 | 1.00(0.77-1.31) | SDM3 | -0.08 | 0.93(0.85-1.01) | SDM3 | 0.03 | 1.03(0.85-1.25) |
|  | SDM5 | 0.08 | 1.08(0.82-1.42) | SDM4 | -0.08 | 0.93(0.70-1.22) | SDM4 | 0.03 | 1.03(0.74-1.42) |
|  | SDM6 | -0.03 | 0.98(0.70-1.35) | SDM6 | -0.10 | 0.90(0.73-1.11) | SDM5 | 0.10 | 1.11(0.90-1.36) |
| Ln(N_  Follow)× | SDM1 | 0.17 | 1.18(0.77-1.81) | SDM1 | 0.22 | 1.24(0.99-1.56) | SDM1 | 0.29 | 1.34(0.94-1.91) |
|  | SDM2 | -0.17 | 0.84(0.53-1.34) | SDM2 | -0.12 | 0.89(0.66-1.19) | SDM2 | -0.04 | 0.96(0.64-1.42) |
|  | SDM3 | -0.03 | 097(0.65-1.43) | SDM3 | 0.02 | 1.02(0.87-1.19) | SDM3 | 0.09 | 1.10(0.80-1.49) |
|  | SDM5 | -0.05 | 0.95(0.63-1.44) | SDM4 | 0.05 | 1.05(0.69-1.59) | SDM4 | 0.13 | 1.13(0.69-1.86) |
|  | SDM6 | -0.13 | 0.88(0.54-1.45) | SDM6 | -0.08 | 0.93(0.66-1.30) | SDM5 | 0.08 | 1.08(0.77-1.51) |
| Ln(N_Post)× | SDM1 | -0.15 | 0.86(0.70-1.06) | SDM1 | -0.13 | 0.88(0.77-0.99)^g^ | SDM1 | 0.07 | 1.07(0.91-1.27) |
|  | SDM2 | -0.23 | 0.79(0.63-0.99)_g_ | SDM2 | -0.21 | 0.81(0.69-0.95)^g^ | SDM2 | -0.01 | 0.99(0.82-1.20) |
|  | SDM3 | -0.07 | 0.93(0.77-1.12) | SDM3 | -0.06 | 0.95(0.87-1.03) | SDM3 | 0.15 | 1.16(1.01-1.34)^g^ |
|  | SDM5 | -0.02 | 0.98(0.80-1.21) | SDM4 | 0.02 | 1.02(0.83-1.25) | SDM4 | 0.22 | 1.25(0.99-1.58) |
|  | SDM6 | -0.22 | 0.80(0.63-1.01) | SDM6 | -0.20 | 0.82(0.70-0.96)^g^ | SDM5 | 0.20 | 1.23(1.04-1.44)^g^ |
| Constant | 15.47 | | | 0.78 | | | 20.31 | | |
